# Investigating mixed pathologies in marmoset models of aging and Alzheimer's disease

**DOI:** 10.1002/alz70855_106132

**Published:** 2025-12-24

**Authors:** Hasi Huhe, Lauren Bailey, Seung‐Kwon Ha, Yongshan Mou, Diego Szczupak, Jung Eun Park, Afonso C Silva, Stacey J Sukoff Rizzo

**Affiliations:** ^1^ University of Pittsburgh School of Medicine, Pittsburgh, PA, USA

## Abstract

**Background:**

Emerging studies indicate that TDP43 and Alpha‐synuclein (αsyn) are often intertwined with Alzheimer's disease (AD) pathologies and are linked to a higher incidence of AD‐related dementia (ADRD). Therefore, animal models of AD with mixed pathology are essential for understanding how these various pathologies may interact and contribute to age‐related cognitive decline, Alzheimer's disease, and dementia. Given the translational limitations of rodent models, the common marmoset (*Callithrix jacchus*) has emerged as an important model system for studying diseases of aging, including Alzheimer's disease (AD). We previously reported the physiological expression of both 3R and 4R tau isoforms in marmosets. Building on our studies in marmosets, we aimed to analyze the age‐related spontaneous presentation of TDP43 and αsyn expression in marmoset brain, and subsequently investigate the interaction of TDP43/αsyn pathology with Aβ/Tau pathology in marmosets with PSEN1 mutations and in those seeded with p301Ltau.

**Method:**

Age‐related spontaneous presentation of TDP43 and αsyn expression were analyzed using Western Blot (WB) and immunohistochemistry (IHC) from brain tissues of unrelated outbred male and female marmosets. Subcellular (nuclear and cytosolic) distribution of TDP43 was examined by WB and visualized by IHC. Aβ/Tau pathology‐related alterations of TDP43 and αsyn were analyzed using brain tissue from marmosets genetically engineered with PSEN1 mutations and those seeded with AAV p301Ltau. Brain TDP43, and plasma Aβ and total Tau were examined by ELISA.

**Result:**

TDP43 and αsyn were expressed in both nuclear and cytosolic fraction across different age groups of outbred marmosets without significant age‐related alterations. IHC revealed the nuclear and cytosolic distribution of physiological expression of TDP43 in the marmoset brain. Ongoing studies are assessing whether TDP43 and αsyn pathologies occur in advanced‐aged marmosets and PSEN1 and tau‐seeded marmosets.

**Conclusion:**

The present studies demonstrate TDP43 and αsyn protein expression and the subcellular distribution in the marmoset brain. These data emphasize the importance of marmoset models for the study of the natural progression of mixed pathologies in aging and ADRD and their association with cognitive decline.

